# Clinical Predictors and Outcomes of Post-transplantation Diabetes Mellitus in an Indian Kidney Transplant Cohort: The Predictive Value of Early Postoperative Hyperglycaemia

**DOI:** 10.7759/cureus.112534

**Published:** 2026-07-12

**Authors:** Pranjal Kashiv, Amit S Pasari, Manish Balwani, Vivek Kute

**Affiliations:** 1 Nephrology, All India Institute of Medical Sciences, Nagpur, Nagpur, IND; 2 Nephrology, Saraswati Kidney Care Center, Nagpur, IND; 3 Nephrology, Jawaharlal Nehru Medical College, Wardha, IND; 4 Nephrology, Institute of Kidney Diseases and Research Centre (IKDRC), Ahmedabad, IND

**Keywords:** indian cohort, kidney transplantation, postoperative hyperglycaemia, post-transplantation diabetes mellitus, risk factors, tacrolimus

## Abstract

Background: Post-transplantation diabetes mellitus (PTDM), formerly termed new-onset diabetes after transplantation, is a major metabolic complication of kidney transplantation that increases cardiovascular risk and jeopardises patient and graft survival. Although well characterised in Western populations, Indian data remain limited, particularly regarding the significance of hyperglycaemia recognised during the index transplant hospitalisation.

Methods: A single-centre retrospective cohort study was conducted of 40 consecutive adult recipients of a first kidney transplant without pre-existing diabetes (May 2023 to December 2024) at a tertiary centre in central India. All patients received tacrolimus-, corticosteroid-, and mycophenolate-based immunosuppression. Inpatient capillary glucose trends, lipid profile, viral serology, and tacrolimus trough concentrations were recorded. PTDM was defined using the American Diabetes Association criteria, applied once patients were clinically stable. Continuous variables were compared using Student's t-test and categorical variables using Fisher's exact test; given the small number of events, analyses were restricted to univariable comparisons.

Results: The cumulative incidence of PTDM was 20% (8/40). Affected recipients were older (42.8 ± 7.5 vs. 28.1 ± 10.1 years; p < 0.001), with higher body mass index (20.3 ± 1.7 vs. 17.4 ± 1.9 kg/m²; p < 0.001) and higher pre-transplant triglycerides (122 ± 26 vs. 98 ± 27 mg/dL; p = 0.03). Postoperative hyperglycaemia occurred in 25.0% of the cohort (10/40) and was strongly associated with PTDM, present in six of eight PTDM recipients (75.0%) versus four of 32 without PTDM (12.5%; p = 0.001). All cases were diagnosed within four months of transplantation, frequently in the setting of tacrolimus troughs >15 ng/mL. Three patients (3/8, 37.5%) had transient and five (5/8, 62.5%) had persistent disease. Graft function and rejection rates were comparable between groups.

Conclusions: PTDM developed in one-fifth of this Indian cohort. Hyperglycaemia detected during the index hospitalisation, together with older age and an adverse pre-transplant metabolic profile, identified recipients at higher risk. Systematic inpatient glucose surveillance offers an actionable window for early intervention, consistent with randomised evidence that early basal-insulin therapy can attenuate progression to sustained PTDM.

## Introduction

Post-transplantation diabetes mellitus (PTDM) is a frequent and consequential metabolic disorder of solid-organ transplantation. The term PTDM, endorsed by successive international consensus meetings in 2003, 2014 and most recently in 2024, has replaced the older designation "new-onset diabetes after transplantation" because it more accurately encompasses both previously undiagnosed diabetes and diabetes arising de novo after grafting [1-3]. Pathophysiologically, PTDM resembles type 2 diabetes, combining tissue insulin resistance with an inadequate β-cell secretory response that is further compromised by calcineurin inhibitor and corticosteroid exposure [4-6].

Reported incidence has varied widely, from roughly 10% to 30% in contemporary series, reflecting heterogeneity in diagnostic criteria, ascertainment methods and follow-up duration [6-8]. The 2024 International Consensus reaffirmed the oral glucose tolerance test as the reference standard for diagnosis and screening, recommended deferral of a formal diagnosis until the patient is clinically stable, typically around six weeks after transplantation, and cautioned against reliance on glycated haemoglobin in the early post-transplant period, when anaemia and dynamic allograft function reduce its sensitivity [1].

Multiple pre-transplant factors contribute to risk, including advancing age, higher body mass index, family history of diabetes, hepatitis C seropositivity, dyslipidaemia, and impaired fasting glucose [1,6,9,10]. Disturbances of glucose metabolism may nonetheless appear in the immediate postoperative period, even in recipients without antecedent diabetes. Whether such inpatient hyperglycaemia is a transient response to surgical stress, glucocorticoids, and intensified immunosuppression, or an early marker of enduring β-cell vulnerability, has important clinical implications. Randomised proof-of-concept and multicentre trials have shown that early basal-insulin therapy initiated in response to perioperative hyperglycaemia can reduce the odds of sustained PTDM, lending mechanistic and therapeutic weight to the early hyperglycaemic signal [11-14].

Against this background, the present study examined a cohort of non-diabetic kidney transplant recipients in central India with three objectives: to determine the incidence of immediate postoperative hyperglycaemia and of subsequent PTDM; to identify factors associated with PTDM; and to evaluate the relationship between perioperative hyperglycaemia and later diabetes, together with its impact on graft function and rejection.

## Materials and methods

Study design and population

A single-centre retrospective cohort study was performed of all adult patients who underwent a first kidney transplant at a tertiary care centre in central India between May 2023 and December 2024. Demographic, clinical, and laboratory data were extracted from hospital records and the electronic database. The study was conducted in accordance with the Declaration of Helsinki and approved by the institutional ethics committee.

Inclusion and exclusion criteria

Eligible patients were first-graft recipients without a history of diabetes mellitus and without prior corticosteroid or other immunosuppressive therapy. Patients with pre-transplant diabetes, previous transplantation, or chronic pre-operative immunosuppressant use were excluded.

Clinical and laboratory parameters

Random, fasting, and post-prandial plasma glucose were recorded for all participants; an oral glucose tolerance test was performed where indicated. Glycated haemoglobin was not used for diagnosis within the first three post-transplant months. Standard biochemistry (urea, creatinine, electrolytes, liver function tests, fasting lipid profile, and urinalysis), viral serology, and 12-hour tacrolimus trough concentrations were obtained. Allograft biopsy was performed when rejection was clinically suspected.

Immunosuppressive protocol

All recipients received a tacrolimus- and corticosteroid-based regimen in accordance with the KDIGO (Kidney Disease: Improving Global Outcomes) recommendations [15]. Tacrolimus (0.10 mg/kg/day) was commenced on the day of transplantation and adjusted to trough concentration, combined with mycophenolate mofetil (2 g/day) or azathioprine (1-2 mg/kg/day). Basiliximab induction (20 mg on days 0 and 4) was given to low-risk living-unrelated recipients; living-related recipients received no induction; and high-risk living-unrelated and all deceased-donor recipients received rabbit antithymocyte globulin (total 5 mg/kg). Perioperative methylprednisolone (1 g intra-operatively, 500 mg on days 1 and 2) was followed by oral prednisolone (0.5 mg/kg/day) tapered over 8-12 weeks to 5-7.5 mg/day. Because all maintenance regimens were uniform, induction type was examined as a covariate.

Definitions

PTDM was diagnosed using the American Diabetes Association criteria, including symptoms of diabetes with a casual plasma glucose ≥200 mg/dL (11.1 mmol/L), or a fasting plasma glucose ≥126 mg/dL (7.0 mmol/L) confirmed on a separate day, applied once the patient was clinically stable, in keeping with the 2024 International Consensus [1]. Inpatient postoperative hyperglycaemia was defined as any capillary glucose ≥200 mg/dL, a requirement for insulin during hospitalisation, or impaired fasting glucose (fasting plasma glucose = 100-125 mg/dL) or impaired glucose tolerance (two-hour plasma glucose = 140-199 mg/dL).

Postoperative management and glucose monitoring

Transplantation was generally performed within seven days of admission. Postoperatively, intravenous crystalloid was matched 1:1 to urine output for the first 24 hours and 0.5:1 thereafter, with 5% dextrose at 100 mL/hour discontinued once oral intake was adequate. Capillary glucose was measured four times daily from day 0. Hyperglycaemia was managed initially with a short-acting insulin sliding scale (commencing at two units, increasing by two units per 50 mg/dL above 200 mg/dL) and escalated to a basal-bolus regimen at the discretion of the treating physician. The monitoring schedule, fluid regimen, and initial insulin sliding scale were applied identically to all recipients; escalation to a basal-bolus regimen and any adjustment of immunosuppression for glycaemic control were physician-directed decisions taken within this standardised framework. Fasting and post-prandial glucose were measured on postoperative days three and seven and before discharge. A composite average of in-hospital glucose values was used to classify each patient as normoglycaemic, hyperglycaemic, or PTDM.

Follow-up

Follow-up followed institutional protocol, with clinical assessment, blood pressure measurement, and laboratory testing at each visit. Glucose was monitored weekly during the first month, fortnightly in the second month, at three, six, and 12 months, and annually thereafter.

Statistical analysis

Continuous variables are presented as mean ± standard deviation and were compared using the independent-samples Student's t-test; categorical variables are presented as n (%) and were compared using the Pearson χ² test or the Fisher exact test, as appropriate, with the corresponding test statistic and degrees of freedom reported alongside each p-value. Given the small number of PTDM events (n = 8), analyses were restricted to univariable comparisons; multivariable modelling was not undertaken, as the events-per-variable ratio was insufficient to support stable adjusted estimates. Accordingly, the reported associations should be interpreted as hypothesis-generating. A two-sided p-value <0.05 was considered statistically significant. Analyses were performed using SPSS version 26 (IBM Corp., Armonk, NY, USA).

## Results

Between May 2023 and December 2024, 40 previously non-diabetic patients underwent kidney transplantation. The cumulative incidence of PTDM was 20% (8/40); 32 recipients (80.0%) did not develop PTDM.

Demographic and clinical characteristics

Recipients who developed PTDM were substantially older than those who did not (42.8 ± 7.5 vs. 28.1 ± 10.1 years; t(38) = 3.84, p < 0.001). Men constituted 75.0% of each group (6/8 vs. 24/32). Smoking (2/8, 25.0% vs. 4/32, 12.5%) and alcohol use (1/8, 12.5% vs. 1/32, 3.1%) were numerically more frequent among patients with PTDM but did not differ significantly (Table 1).

**Table 1:** Clinical and demographic characteristics of kidney transplant recipients stratified by PTDM status. Values are presented as n (%) unless otherwise indicated. Continuous variables were compared using the independent-samples Student's t-test, with the test statistic and degrees of freedom shown (t(df)). Categorical variables were compared using the Fisher exact test, as expected cell counts were <5 in all categorical comparisons; two-sided p-values are reported. A two-sided p < 0.05 was considered statistically significant. BMI, body-mass index; CMV, cytomegalovirus; D, donor; HBsAg, hepatitis B surface antigen; HCV, hepatitis C virus; PTDM, post-transplantation diabetes mellitus; R, recipient; SD, standard deviation.

Characteristic	Total (n = 40)	No PTDM (n = 32)	PTDM (n = 8)	Test statistic	P-value
Age, years (mean ± SD)	33.9 ± 10.6	28.1 ± 10.1	42.8 ± 7.5	t(38) = 3.84	<0.001
Male sex	30 (75.0)	24 (75.0)	6 (75.0)	—	1.00
Female sex	10 (25.0)	8 (25.0)	2 (25.0)	—	1.00
Current smoker	6 (15.0)	4 (12.5)	2 (25.0)	—	0.58
Alcohol use	2 (5.0)	1 (3.1)	1 (12.5)	—	0.36
Donor type — living related	19 (47.5)	15 (46.9)	4 (50.0)	—	1.00
Donor type — living unrelated	13 (32.5)	11 (34.4)	2 (25.0)	—	1.00
Donor type — deceased	8 (20.0)	6 (18.8)	2 (25.0)	—	0.65
Donor age, years (mean ± SD)	43.4 ± 6.2	44.0 ± 6.4	41.0 ± 5.9	t(38) = 1.20	0.24
Male donor	16 (40.0)	12 (37.5)	4 (50.0)	—	0.69
Female donor	24 (60.0)	20 (62.5)	4 (50.0)	—	0.69
Tacrolimus	40 (100)	32 (100)	8 (100)	—	—
Corticosteroids	40 (100)	32 (100)	8 (100)	—	—
Mycophenolate mofetil	40 (100)	32 (100)	8 (100)	—	—
β-blocker	19 (47.5)	16 (50.0)	3 (37.5)	—	0.70
Thiazide diuretic	1 (2.5)	1 (3.1)	0 (0)	—	1.00
Haemodialysis	38 (95.0)	30 (93.8)	8 (100)	—	1.00
Peritoneal dialysis	2 (5.0)	2 (6.2)	0 (0)	—	1.00
Dialysis vintage, months (mean ± SD)	10.4 ± 6.4	10.4 ± 5.2	10.2 ± 9.8	t(38) = 0.08	0.94
Dialysis <12 months	29 (72.5)	23 (71.9)	6 (75.0)	—	1.00
Dialysis ≥12 months	11 (27.5)	9 (28.1)	2 (25.0)	—	1.00
BMI, kg/m² (mean ± SD)	17.7 ± 2.5	17.4 ± 1.9	20.3 ± 1.7	t(38) = 3.93	<0.001
Triglycerides, mg/dL (mean ± SD)	104 ± 27	98 ± 27	122 ± 26	t(38) = 2.26	0.03
HCV seropositive (pre-transplant)	0 (0)	0 (0)	0 (0)	—	—
HBsAg positive	3 (7.5)	2 (6.2)	1 (12.5)	—	0.50
CMV D+/R+	35 (87.5)	28 (87.5)	7 (87.5)	—	1.00
CMV D–/R+	1 (2.5)	1 (3.1)	0 (0)	—	1.00
CMV D+/R–	2 (5.0)	1 (3.1)	1 (12.5)	—	0.36
CMV D–/R–	2 (5.0)	2 (6.2)	0 (0)	—	1.00
Delayed graft function	4 (10.0)	3 (9.4)	1 (12.5)	—	1.00
Acute rejection (biopsy-proven)	5 (12.5)	4 (12.5)	1 (12.5)	—	1.00

Donor and transplant characteristics

Most recipients received living-donor grafts. Donor type, donor age, and donor sex did not differ between groups, and the majority of donors were female (24/40, 60.0%). Pre-transplant dialysis vintage was similar (10.4 ± 5.2 vs. 10.2 ± 9.8 months), with almost all patients managed by haemodialysis (38/40, 95.0%) (Table 1).

Metabolic and viral parameters

Mean body mass index was higher in the PTDM group (20.3 ± 1.7 vs. 17.4 ± 1.9 kg/m²; t(38) = 3.93, p < 0.001), as were pre-transplant triglycerides (122 ± 26 vs. 98 ± 27 mg/dL; t(38) = 2.26, p = 0.03). Three recipients (3/40, 7.5%) were hepatitis B surface antigen (HBsAg) positive, one of whom (1/8, 12.5%) developed PTDM. No recipient was hepatitis C virus (HCV) seropositive before transplantation, although one acquired HCV thereafter. Cytomegalovirus serostatus was predominantly D+/R+ in both groups (35/40, 87.5%) (Table 1).

Postoperative hyperglycaemia

Postoperative hyperglycaemia occurred in 10 of 40 recipients (25.0%). It was present in six of eight patients who developed PTDM (75.0%) compared with four of 32 who did not (12.5%), a strong and statistically significant association (Fisher exact test, p = 0.001) (Table 2).

**Table 2:** Association between inpatient postoperative hyperglycaemia and subsequent PTDM. Values are presented as n (%). The association was assessed using the Pearson χ² test (χ²(1) = 13.33, p < 0.001). Because two cells had expected counts <5, the Fisher exact test was applied as the primary confirmatory analysis (p = 0.001). Both tests indicated a statistically significant association at the predefined threshold of p < 0.05. PTDM, post-transplantation diabetes mellitus; df, degrees of freedom.

Postoperative hyperglycaemia	No PTDM (n = 32)	PTDM (n = 8)	Total (n = 40)	χ² (df)	P-value
Absent	28 (87.5)	2 (25.0)	30 (75.0)	13.33 (1)	<0.001
Present	4 (12.5)	6 (75.0)	10 (25.0)	—	—
Total	32 (100)	8 (100)	40 (100)	—	—

Interval to diagnosis and tacrolimus exposure

All patients with PTDM (8/8, 100%) were receiving tacrolimus-based immunosuppression and consistently had high tacrolimus troughs (>15 ng/mL) around the time of diagnosis. The interval from transplantation to onset of hyperglycaemia ranged from one to 97 days (mean = 34 days), with every case identified within four months.

Risk-factor analysis

On univariable comparison, older recipient age, higher pre-transplant triglycerides, higher body mass index, and inpatient postoperative hyperglycaemia were each significantly associated with PTDM (Tables 1, 2). Given the limited number of events (eight cases), multivariable modelling was not undertaken, as the events-per-variable ratio was insufficient to support stable adjusted estimates; the associations reported here are therefore univariable and should be interpreted as hypothesis-generating.

Treatment, course, and graft outcomes

Among the eight patients with PTDM, three (3/8, 37.5%) required inpatient management of severe hyperglycaemia with insulin. Immunosuppression was adjusted to assist glycaemic control in most patients. Four patients (4/8, 50.0%) ultimately required insulin (alone or with an oral agent), one (1/8, 12.5%) was maintained on oral monotherapy, and three (3/8, 37.5%) achieved control through immunosuppressive modification alone. Three patients (3/8, 37.5%) experienced resolution and were classified as transient PTDM, whereas five (5/8, 62.5%) had persistent disease; transient disease predominated among men and persistent disease among women. Renal function remained stable, with serum creatinine of 0.9-2.1 mg/dL and Chronic Kidney Disease Epidemiology Collaboration (CKD-EPI) estimated glomerular filtration rate (eGFR) of 41-76 mL/min/1.73 m² over six to 21 months of follow-up. Delayed graft function and biopsy-proven acute rejection occurred at similar rates in both groups (Table 1).

## Discussion

In this Indian cohort, PTDM developed in one-fifth of non-diabetic kidney transplant recipients, and hyperglycaemia recognised during the index hospitalisation emerged as the factor most strongly associated with its development. This finding aligns with a growing body of evidence that the perioperative glycaemic trajectory carries prognostic information well beyond the acute setting [5,11].

Kidney transplantation remains the optimal treatment for end-stage kidney disease, yet PTDM erodes its benefit by increasing cardiovascular morbidity and impairing patient and graft survival [9,10,16-18]. The disorder also imposes a substantial economic burden [17]. Most earlier studies ascertained PTDM only after discharge [9,19,20]; by contrast, the Vienna group demonstrated in successive randomised trials that early basal-insulin therapy, triggered by afternoon hyperglycaemia, can reduce the odds of sustained PTDM by approximately three-quarters in the proof-of-concept study and more modestly in the larger multicentre trial, albeit at the cost of more frequent hypoglycaemia [12-14]. A subsequent trial of continuous subcutaneous insulin infusion did not show superiority over basal insulin or standard care, underscoring that the optimal modality remains unsettled even as the principle of early intervention is reinforced [14]. The present observation that 25.0% of recipients developed inpatient hyperglycaemia, and that this was strongly associated with subsequent PTDM, supports systematic perioperative glucose surveillance as a pragmatic risk-stratification tool.

Age was again confirmed as a determinant of risk, with affected recipients markedly older than unaffected ones, consistent with prior reports of a two- to three-fold increase in older patients [6,9,10]. No significant association was found with recipient or donor sex, donor relationship, or dialysis modality and vintage, in keeping with the literature [20,21]. Although higher body mass index and triglycerides characterised the PTDM group, the small number of events limited the statistical power available to evaluate these variables in adjusted analyses. The notably low mean body mass index in both groups reflects the lean, often undernourished phenotype of the Indian dialysis population and should be interpreted in that context.

Tacrolimus exposure is a well-established, dose-dependent contributor to PTDM through impaired insulin secretion [5,11,22]. All patients with PTDM had troughs exceeding 15 ng/mL around diagnosis, reinforcing the value of avoiding supratherapeutic exposure; the 2024 consensus and contemporary reviews advocate tacrolimus minimisation, judicious corticosteroid reduction and consideration of belatacept-based regimens in selected high-risk recipients [1,5,23]. No association was observed between PTDM and acute rejection, and graft function was preserved across groups during follow-up.

The therapeutic landscape has shifted appreciably. Beyond insulin in the acute phase, sodium-glucose cotransporter-2 inhibitors and glucagon-like peptide-1 receptor agonists are increasingly used in stable transplant recipients, with accumulating evidence of glycaemic, weight, and cardiorenal benefit and an acceptable safety profile, although randomised data specific to PTDM remain limited and genitourinary infection warrants vigilance with SGLT2 inhibition [1,5,24]. Lifestyle intervention, though not formally tested in transplant populations, is a reasonable extrapolation from diabetes-prevention trials in the general population and is readily initiated during hospitalisation, when structured education is feasible [1].

This study has limitations. It is retrospective, single-centre, and modest in size, with only eight PTDM events. The small number of events precluded stable multivariable modelling, so the reported associations are univariable and should be regarded as hypothesis-generating; residual confounding cannot be excluded. Routine oral glucose tolerance testing, the consensus reference standard, was not performed, which may have led to underascertainment of milder dysglycaemia. Family history of diabetes was incompletely captured. These constraints temper causal inference but do not negate the consistent, biologically plausible signal linking early hyperglycaemia to PTDM.

## Conclusions

Inpatient postoperative hyperglycaemia is a strong, readily measurable correlate of PTDM after kidney transplantation in this Indian cohort, alongside older age and an adverse pre-transplant metabolic profile. As the perioperative period offers a defined window for surveillance and early therapy, and because randomised evidence supports early intervention against perioperative hyperglycaemia, routine inpatient glucose monitoring should be regarded as an integral component of transplant care. Larger, prospective, oral glucose tolerance test-anchored studies are needed to confirm these associations, to elucidate the mechanisms linking early hyperglycaemia to sustained PTDM, and to define optimal preventive strategies in high-risk recipients.
